# CSF p-tau increase in response to Aβ-type and Danish-type cerebral amyloidosis and in the absence of neurofibrillary tangles

**DOI:** 10.1007/s00401-021-02400-5

**Published:** 2021-12-28

**Authors:** Stephan A. Kaeser, Lisa M. Häsler, Marius Lambert, Carina Bergmann, Astrid Bottelbergs, Clara Theunis, Marc Mercken, Mathias Jucker

**Affiliations:** 1 Department of Cellular Neurology, Hertie Institute for Clinical Brain Research, University of Tübingen, 72076 Tübingen, Germany; 2 DZNE, German Center for Neurodegenerative Diseases, 72076 Tübingen, Germany; 3 Neuroscience Department, Janssen Research and Development, Beerse, Belgium

Phosphorylated tau (p-tau) species in bodily fluids are among the most reliable molecular biomarkers for differential diagnosis and progression monitoring of Alzheimer’s disease (AD) [3]. The ATN research framework stages AD patients based on three classes of readouts, amyloid (A), tau (T) and neurodegeneration (N) including cerebrospinal fluid (CSF) and imaging biomarkers [6]. CSF p-tau together with positron emission tomography (PET) for tau are suggested biomarkers of tau pathology. While tau PET is clearly related to tauopathy by tracing brain neurofibrillary tangles, for p-tau it is less clear whether it reflects or rather anticipates early tangle formation. Several studies could show that the increase of fluid-based tau phosphorylated at threonine 181 (p181tau) and tau phosphorylated at threonine 217 (p217tau) is an early event of AD pathogenesis driven by β-amyloid deposition in brain [1, 3]. The tight link between β-amyloid deposition and CSF p-tau is also consistent with recent clinical data using anti-Aβ antibody Donanemab; concomitant with a reduction in Aβ plaques, plasma p217tau decreased while tauopathy still progressed, although at a slower rate [8]. However, whether β-amyloidosis per se, i.e. in the absence of neurofibrillary tangles and neuronal death, is sufficient to raise p-tau levels in the CSF is not clear [3].

We quantified endogenous p181tau in CSF samples of APPPS1 transgenic (tg) mice (see also Supplementary Methods, online resource) that do not develop neurofibrillary tangles or extensive neuron loss [9]. CSF p181tau in APPPS1 tg mice showed an age-dependent increase reaching a plateau at three- to four-fold higher levels in aged compared to 1.5-month-old mice (Fig. 1a). In non-tg littermates, CSF p181tau exhibited a biphasic profile with a transient drop reminiscent of CSF total tau (t-tau) levels in non-tg mice [10]. Total tau (t-tau) measured in the same CSF samples also plateaued in aged APPPS1 tg mice (Supplementary Fig. 1, online resource). The p181tau/t-tau ratio initially dropped but overall remained stable at 7–8% (Fig. 1b). CSF p181tau strongly correlated with CSF t-tau levels (Fig. 1c).

**Fig. 1:**
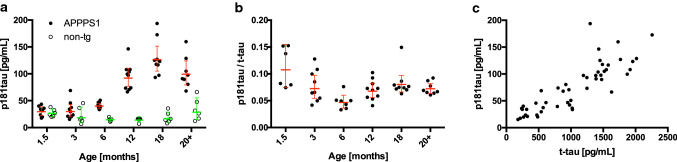
APPPS1 mice exhibit age-dependent increase of p181tau in the CSF. Male and female 1.5, 3, 6, 12, 18 and 20–22-month-old APPPS1 (8–10 per group) and non-tg littermates (5–6 per group) were used to assess CSF p181tau and t-tau. **a** Two-way analysis of variance (ANOVA) revealed a significant age × genotype interaction (*F*[5, 77] = 19.8; *p* < 0.0001). In APPPS1 mice, CSF p181tau was significantly increased after 12 months compared to the youngest group, however, at 6 months there was already a difference in CSF-p181tau between APPPS1 and non-tg littermates (Tukey post hoc test *p* < 0.0001 and *p* = 0.0029, respectively). **b** One-way ANOVA revealed a significant age effect on p181tau/t-tau ratio in APPPS1 mice (*F*[5, 43] = 5.4, *p* = 0.0006). For t-tau see Supplementary Fig. 1, online resource. After an initial decrease between 1.5 and 6 months of age (Tukey test, *p* < 0.0001) p181tau/t-tau increased again between 6 and 18 months (Tukey test, *p* < 0.0131) but stagnated thereafter. Shown are the geometric means ± confidence interval; statistics in **a** and **b** are based on log transformed values. **c** Relationship between CSF p181tau and t-tau showed a strong positive correlation (Spearman rank correlation test: *ρ* = 0.86, *p* < 0.0001)

We then used an immunoassay which allows the quantification of tau phosphorylated at threonine 217 with or without adjacent phospho-epitopes (“p217 + tau” [12] (see also Supplementary Methods, online resource). P217 + tau also showed an age-dependent increase and reached a plateau, although at 14- to 16-fold higher levels compared to 1.5-month-old APPPS1 tg animals (Fig. 2a). The p217 + tau/t-tau ratio was unchanged up to 6 months of age but started to increase thereafter (from 5 to 14%) becoming significant at 18 months of age (Fig. 2b). A strong correlation was observed between CSF p217 + tau and t-tau levels (Fig. 2c).

**Fig. 2:**
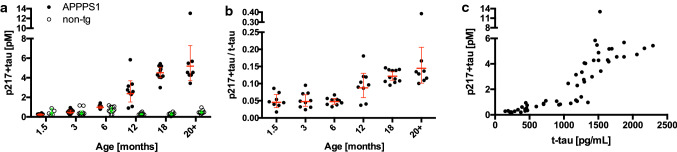
APPPS1 mice exhibit an age-dependent increase of p217 + tau in the CSF. Male and female APPPS1 at the age of 1.5, 3, 6, 12, 18 and 20–22 months (8–11 mice/group) and non-tg littermates (5–10 mice/group) were used. This is a new cohort of mice and different from the one shown in Fig. 1. **a** Two-way ANOVA revealed a significant age × genotype interaction (*F*[5, 95] = 41.7; *p* < 0.0001). CSF p217 + tau was significantly increased starting from 6 months on compared to the youngest group, however, only at 12 months of age there was a significant difference between APPPS1 and non-tg littermates (Tukey post hoc test, *p* < 0.0001 for both). **b** One-way ANOVA revealed a significant age effect on p217 + tau/t-tau ratio in APPPS1 mice (*F*[5, 48] = 16.5, *p* < 0.0001). For t-tau see Supplementary Fig. 1, online resource. CSF p217 + tau/t-tau increased between 6 and 12 months of age (Tukey test, *p* = 0.0043) reaching an apparent plateau. Shown are the geometric means ± confidence interval; statistics in **a** and **b** are based on log transformed values. **c** The relationship between CSF p217 + tau and t-tau showed a strong positive correlation (Spearman rank correlation test: *ρ* = 0.94, *p* < 0.0001)

Thus, overall changes in CSF p181tau, p217 + tau and t-tau tightly follow the Aβ deposition reported in this mouse model starting at 1.5 months with a plateau around 18 months of age [9, 10, 13]. The magnitude of the CSF p-tau increase is comparable to the p-tau increase observed in AD patients [7]. In AD, soluble p-tau also reaches its highest level in the phase of maximal cerebral amyloid load, but seems to decrease thereafter, presumably due to the occurrence of neuron loss during disease progression [1].

To test whether the increase of CSF p-tau is specific to the aggregation of Aβ or rather a shared consequence of different types of cerebral amyloidosis, we then assessed tau in the CSF of ADanPP tg mice, a model of Danish amyloidosis as seen in Familial Danish Dementia (FDD) [2, 5] (see also Supplementary Methods, online resource). Again, a marked age-related increase of CSF p217 + tau was observed, which was absent in non-tg control mice (Fig. 3a). On average, aged ADanPP tg mice had up to 43-fold higher CSF p217 + tau levels compared to 3-month-old tg mice. Endogenous t-tau revealed a seven- to eightfold increase in aged compared to young ADanPP tg mice similar to the six- to sevenfold increase observed in APPPS1 tg mice (Supplementary Fig. 1, online resource). The ratio of CSF p217 + tau/t-tau raised from 8 to 25% (Fig. 3b). CSF p217 + tau and t-tau levels in ADanPP tg mice were again strongly correlated (Fig. 3c).

**Fig. 3:**
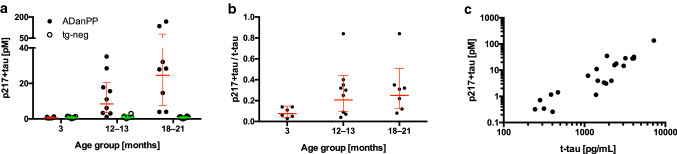
ADanPP mice exhibit an age-dependent increase of p217 + tau in the CSF. Male and female ADanPP and non-tg littermates at the age of 3, 12–13, and 18–21 months (6–9 mice/group) were used. **a** Two-way ANOVA revealed a significant age × genotype interaction (*F*[2, 37] = 15.4; *p* < 0.0001). At 12–13 months of age, CSF p217 + tau was already significantly increased compared to the youngest group and age-matched non-tg littermates (Tukey post hoc test, *p* < 0.0001 and *p* = 0.0008, respectively). **b** One-way ANOVA revealed a significant age effect on p217 + tau/t-tau ratio (*F*[2, 19] = 18.15, *p* < 0.0001) with significant increases in the 12–13- and 18–20-month-old ADanPP mice compared to the youngest age group (Tukey test, *p* = 0.0004 and *p* < 0.0001, respectively; note that one value > 1 in the oldest age group was excluded from the statistical analysis because p217 + tau values above 100% of t-tau are not scientifically reasonable). For t-tau see Supplementary Fig. 1, online resource. Shown are the geometric means ± confidence interval, statistics in **a** and **b** are based on log transformed values. **c** The relationship between CSF p217 + tau and t-tau showed a strong positive correlation (Spearman rank correlation test: *ρ* = 0.89, *p* < 0.0001). Log10 scale was used on *x*- and *y*-axis

These observations imply that p-tau increases in CSF are not exclusively triggered by Aβ deposition but can also be induced by the deposition of Danish amyloid (ADan). Thus, it is tempting to speculate that CSF p-tau increases are a general phenomenon of secondary tauopathies, as opposed to primary tauopathies. Interestingly, AD and FDD but also Familial British Dementia, yet another cerebral amyloidosis with concomitant neurofibrillary degeneration [4]), all share ultrastructural commonalities of tau filaments [11]. Overall, the present results support a more differentiated assignment of fluid-based molecular tau species in the ATN framework especially at early disease stages where therapeutic interventions are most promising.

## Supplementary Information

Below is the link to the electronic supplementary material.

Supplementary file1 (DOCX 101 kb)
