# Supplementary material for: CSF p-tau increase in response to Aβ-type and Danish-type cerebral amyloidosis and in the absence of neurofibrillary tangles
Source: Acta Neuropathol. 2021 Dec 28;143(2):287–90. doi: 10.1007/s00401-021-02400-5 (PMC8742811; doi:10.1007/s00401-021-02400-5)
Supplement: Supplementary file 1 — Supplementary file1 (DOCX 101 kb) [file 401_2021_2400_MOESM1_ESM.docx]

**ONLINE RESOURCE**

**CSF p-tau increase in response to Aβ-type and Danish-type cerebral amyloidosis and in the absence of neurofibrillary tangles**

Stephan A. Kaeser^1,2^, Lisa M. Häsler^1,2^, Marius Lambert^1,2^, Carina Bergmann^1,2^, Astrid Bottelbergs^3^, Clara Theunis^3^, Marc Mercken^3^, Mathias Jucker^1,2^

^1^Department of Cellular Neurology, Hertie Institute for Clinical Brain Research, University of Tübingen, 72076 Tübingen, Germany; ^2^DZNE, German Center for Neurodegenerative Diseases, 72076 Tübingen, Germany; ^3^Neuroscience Department, Janssen Research and Development, Beerse, Belgium

**Correspondence**: Stephan Kaeser, stephan.kaeser@uni-tuebingen.de; or Mathias Jucker, mathias.jucker@uni-tuebingen.de. Phone: +49 7071 29 86863; Fax: +49 7071 29 4521

**SUPPLEMEMNTARY METHODS**

**APPPS1 mice**

Male and female 1.5- to 22-month-old APPPS1 mice [4] and gender and age-matched non-transgenic control mice were bred at the Hertie Institute for Clinical Brain Research. Sex was balanced in all the groups used and no difference between male and female was found. APPPS1 mice have initially been generated and are maintained on a C57BL/6 background and co-express K670M/N671L mutated amyloid precursor protein (APP) and L166P mutated presenilin 1 (PS1) under the control of a neuron-specific Thy1 promoter element. The mice develop first Aβ plaques after 6 weeks of age, and no effect of gender was found [4]. All mice were kept under specific pathogen-free conditions. The experimental procedures were undertaken in accordance with the veterinary office regulations of Baden-Württemberg (Germany) and approved by the local Animal Care and Use Committees.

**ADanPP mice**

Male and female ADanPP mice (3, 12–13 and 18–20 months of age) were bred at the Hertie Institute for Clinical Brain Research. Sex was balanced in all the groups used and no difference between male and female was found. They were generated and maintained on a C57BL/6 background and overexpress human BRI2 (ITM2B) with the Danish mutation (i.e. 10 base pair insert at position 795, TTTAATTTGT) under the control of the cosmid-based Syrian Hamster prion protein expression cassette. First amyloid deposits occur in the hippocampus and in meningeal vessels at 2 months of age and show an age-related increase [1]. All mice were kept under specific pathogen-free conditions. The experimental procedures were in accordance with the veterinary office regulations of Baden-Württemberg (Germany), and were approved by the local Animal Care and Use Committees.

**CSF collection and tissue harvesting**

CSF collection has been described previously [3, 5]. Briefly, mouse was deeply anesthetized with ketamine (100 mg/mL) xylazine (10 mg/mL) and kept on a heating pad during the whole procedure to avoid hypothermia. Surgery was done under a dissecting microscope. The skin was opened by an incision from the bregma to the occipital bone and fixed by means of a colibri retractor. Underlying tissue and neck muscles were separated bluntly and kept apart using a retraction system (Fine Science Tools) in order to expose the cisterna magna with the overlaying dura. For CSF collection, the mouse was kept in a horizontal position and the head was bent over (approx. 45 °) but not fixed. The dura was carefully perforated with a 30-gauge needle and the CSF was collected with a GELoader Tip (Eppendorf) shortened by about 2 cm at the tip. CSF samples were immediately centrifuged at 2,000x g for 10 minutes at room temperature, assessed macroscopically for blood contamination, aliquoted (5 µL) into polypropylene tubes (Eppendorf), snap-frozen in liquid nitrogen, and stored at -80 °C until use.

**Ultrasensitive sandwich immunoassay for t-tau and p-tau measurements**

For the measurement of murine total tau a bead-based immunoassay was developed using Single Molecule Array (Simoa) technology (Quanterix, Lexington, MA, USA), as described previously [5]: In brief, the capture antibody (ADx202; ADx NeuroSciences, Gent, Belgium) was immobilized on paramagnetic microparticles and the detection antibody (77E9; BioLegend, San Diego, CA, USA) was biotinylated following standard procedures. Recombinant murine tau-430 (gift of E.M. Mandelkow, Bonn, Germany) was used as calibrator protein. Samples were processed using a 2-step assay protocol and measured at a final dilution of 1:60 in duplicates on a HD-1 Analyzer (Quanterix). A weighted (1/Y²) 4-parametric logistics was used for curve fitting and calculation of tau protein content in mouse samples.

P217tau concentrations were determined using an assay from Janssen R&D (La Jolla, CA, USA) named p217+tau “short” [6]. The anti-p-tau capture antibody in this assay (PT3) showed strongest binding to tau species encompassing the epitope phosphorylated at threonines 212 and 217, designated as “p217+tau” [7]. The anti-total tau antibody PT82 (Janssen R&D) was used as detection antibody. The assay was calibrated using a 4.5 kDa synthetic peptide containing the human tau (2N4R) sequence 119–126 (the binding epitope of PT82) and 210–220 fused by a PEG4 linker. Since the standard calibrator is smaller than full-length tau, p217+tau levels are reported in pM or had to be normalized to full-length tau. A dilution of 1:25 was applied to the samples and assays were performed in duplicate measurement on a HD-1 or HD-X Analyzer (note: due to low volume, p217+tau levels in 14 CSF samples of the ADanPP mouse cohort could only be measured in singlicates with a 1:30 dilution). For the measurement of p181tau the commercial Simoa p-tau-181 Advantage Kit (version 1, Catalog number 103377, Quanterix) was used. Samples were diluted 1:25 in the provided sample diluent and measured in duplicates on a SR-X System (Quanterix) according to manufacturer’s instructions. For all assays performed, internal reference samples were used as controls on every plate. Specificity of assays was confirmed with CSF samples of tau-deficient mice [2].

**Statistical Analysis**

The distribution of quantitative data was assessed with Shapiro-Wilk test. Outliers were identified by Grubb’s test (in the APPPS1 cohorts) or ROUT test (in the ADanPP cohort; Q=0.1%). Non-normally distributed variables were logarithmic-transformed. To examine whether CSF t-tau and p-tau levels change with aging ANOVA analysis was performed. Differences were analyzed using Tukey’s post hoc test for multiple comparisons. In all cases statistical significance was set at p < 0.05. Graphpad Prism version 6 was used to generate the graphics, and JMP version 15 was used for statistical analysis. If the measured individual values were below the assay’s lower limit of quantification (LLoQ), a fixed value (LLoQ/­√2) was imputed.

**References**

1 Coomaraswamy J, Kilger E, Wolfing H, Schafer C, Kaeser SA, Wegenast-Braun BM, Hefendehl JK, Wolburg H, Mazzella M, Ghiso Jet al (2010) Modeling familial Danish dementia in mice supports the concept of the amyloid hypothesis of Alzheimer's disease. Proc Natl Acad Sci U S A 107: 7969-7974 Doi 10.1073/pnas.1001056107

2 Dawson HN, Ferreira A, Eyster MV, Ghoshal N, Binder LI, Vitek MP (2001) Inhibition of neuronal maturation in primary hippocampal neurons from tau deficient mice. J Cell Sci 114: 1179-1187

3 Maia LF, Kaeser SA, Reichwald J, Lambert M, Obermuller U, Schelle J, Odenthal J, Martus P, Staufenbiel M, Jucker M (2015) Increased CSF Abeta during the very early phase of cerebral Abeta deposition in mouse models. EMBO Mol Med 7: 895-903 Doi 10.15252/emmm.201505026

4 Radde R, Bolmont T, Kaeser SA, Coomaraswamy J, Lindau D, Stoltze L, Calhoun ME, Jaggi F, Wolburg H, Gengler Set al (2006) Abeta42-driven cerebral amyloidosis in transgenic mice reveals early and robust pathology. EMBO reports 7: 940-946 Doi 10.1038/sj.embor.7400784

5 Schelle J, Hasler LM, Gopfert JC, Joos TO, Vanderstichele H, Stoops E, Mandelkow EM, Neumann U, Shimshek DR, Staufenbiel Met al (2017) Prevention of tau increase in cerebrospinal fluid of APP transgenic mice suggests downstream effect of BACE1 inhibition. Alzheimers Dement 13: 701-709 Doi 10.1016/j.jalz.2016.09.005

6 Triana-Baltzer G, Van Kolen K, Theunis C, Moughadam S, Slemmon R, Mercken M, Galpern W, Sun H, Kolb H (2020) Development and Validation of a High Sensitivity Assay for Measuring p217 + tau in Cerebrospinal Fluid. Journal of Alzheimer's disease : JAD 77: 1417-1430 Doi 10.3233/JAD-200463

7 Van Kolen K, Malia TJ, Theunis C, Nanjunda R, Teplyakov A, Ernst R, Wu SJ, Luo J, Borgers M, Vandermeeren Met al (2020) Discovery and Functional Characterization of hPT3, a Humanized Anti-Phospho Tau Selective Monoclonal Antibody. Journal of Alzheimer's disease : JAD 77: 1397-1416 Doi 10.3233/JAD-200544

**SUPPLEMENTARY FIGURE**

**Supplementary Fig. 1.** *Age-dependent increase of CSF t-tau in APPPS1 and ADanPP tg mice.* (**a**) Shown are the CSF t-tau levels of the combined mouse cohorts from Fig. 1 and Fig. 2. One-way ANOVA revealed a significant age effect (F[5, 97] = 95.6; P<0.0001). The first significant increase of CSF t-tau was observed at 6 months of age compared to the youngest age group (Tukey test p<0.0001). Although there was still an increase between 12 and 18 months (Tukey test p=0.0005), CSF t-tau concentration appeared to plateau in aged mice. (**b**) CSF samples from the same mice as in Fig. 3. were used to assess t-tau levels. Two-way ANOVA revealed a significant age x transgene effect (F[2, 37] = 29.2; P<0.0001). In 12- to13-month-old ADanPP mice t-tau levels were significantly increased compared to 3-month-old ADanPP or non-transgenic mice (Tukey test: p<0.0001 for both). Shown are mean (±SEM) in (a) while the geometric means of each group (± confidence interval) is shown in (b) because of non-normal distributed values.
